# Rate of Family Refusal of Organ Donation in Dead-Brain Donors: the Iranian Tissue Bank Experience

**Published:** 2013-05-01

**Authors:** M. Mahdavi-Mazdeh, A. Khodadadi, N. Tirgar, N. Riazi

**Affiliations:** 1 Iranian Tissue Bank and Research Center, Tehran University of Medical Sciences, Tehran, Iran; 2 Nephrology Research Center, Tehran University of Medical Sciences, Tehran, Iran

**Keywords:** Organ donation, Brain death, Transplantation

## Abstract

**Background:** The growing gap between organ supply and demand remains a worldwide serious problem. Losing dead-brain donor organs can be attributed to several reasons including un-recognition of potential donor by ICU staff, death before official declaration of brain death and high refusal rate of deceased donors' families.

**Objective:** To study the trend of dead-brain patients' relatives refusal of organ donation from 2007 to 2011.

**Methods:** This study was a retrospective review of all patients who had been introduced as brain death to the organ procurement unit (OPU) of Iranian Tissue Bank between April 2007 and April 2012 according to preliminary neurological exam performed in the hospital of origin. The refusal rate of dead-brain patients' families and its reasons was evaluated.

**Results:** A total of 874 ICU admitted patients with severe brain injury (Glasgow coma score <7) was introduced to our center and were visited by the coordinator team during April 2007 to April 2012. 412 (47%) patients were excluded from the study mainly due to unsuitability for donation according to the approved medical protocols (n=205) and not fulfilling the brain death criteria (n=66). The families of the remaining cases (n=462) had been interviewed 343 (74.2%) of whom permitted donation.

The mean±SD age of donors was 29.8±13.2 years; the male/female ratio was almost 2. The most common reason of brain death was traffic collision (n=120; 56.3%) and cerebrovascular accidents (n=40; 18.8%). The refusal rate from 2007 to 2011 has decreased respectively, from 30.4% to 20% in Tehran, and from 57.1% to 51.6% in other cities. The overall refusal rate was 25.8%.

**Conclusion: **Our study confirmed that more level of expertise of the coordinator team and continuous public education, would result in higher rate of consent to organ donation.

## Introduction

The growing gap between organ supply and demand remains a worldwide serious problem for patients with end-stage organ failure. Each country has tried various models to tackle this issue [1, 2]. The United States achieved an increase in deceased transplants by extended use of older donors (23 pmp in 2000 compared to 25.6 pmp in 2010), as the motor vehicle crash deaths decreased significantly by enforcing the comprehensive road safety laws [3, 4]. It is reported that over 90% of the world’s fatalities on the roads take place in low- and middle-income countries (21.5 and 19.5 per 100,000 population, respectively). This rate in Iran is 32.7 per 100,000 population, annually [5]. It seems that if the donor coordinator team could act quickly and approach correctly the family of one whose life is not salvageable, other lives might be saved through transplantation of the patient’s organs.

After the legislation of “organ transplantation and brain death act” ratified in 2000 and allocation of a large amount of budget to organ procurement units (OPU) and hospitals by the Ministry of Health and Medical Education, the brain death donation (BDD) program has been supported and deceased donation has increased from 0.2 pmp in 2000 to 4.1 pmp in 2010 [3]. However, the program is still in its infancy and we need more than 3-fold this rate to save patients' lives.

One of the reasons for losing such organs is not to recognizing potential donors by ICU staff, announcing death of patient before official declaration of brain death and a high refusal rate of deceased donors' families to donate organs [4, 6].

In Iran, the BDD network consists of 13 OPUs. There are also five brain death identification units in cities without any transplantation centers that refer cases to the OPUs. Donor transfer occurs by the coordinating team and donor’s medical condition is managed untill harvesting [1, 7]. Regions without OPU are covered by nearby OPUs.

We conducted this study to determine the trend of dead-brain patients' relatives refusal to donate patients’ organs since 2007 to 2011.

## Patients and Methods:

This study was a retrospective review of all patients who had been introduced as brain death to the OPU of Iranian Tissue Bank between April 2007 and April 2012, according to a preliminary neurological exam performed in the hospital of origin. The refusal rate of dead-brain patients' families to donate the patients’ organs and their reasons were evaluated.

According to the Iranian national routines, being informed of a potential dead-brain donor, the brain death identification unit staff goes to the hospital, approaches the family; if the family members agree to donate the patient’s organ, the management process to save organs and maintain their suitability and preliminary tests, verbal consultation with specialists starts and after a satisfactory electroencephalogram (EEG), the identified dead-brain donor is transferred to the center ICU. After admission, parallel with medical supervision and monitoring to confirm brain death to declare it officially, the family gives their written approval.

The obtained data of patients from transplant coordinator team consisted of age, sex, cause of death, duration of ICU admission to the first positive neurological examination indicating brain death, the duration from diagnosis to transfer to OPU and confirmatory tests for the declaration of brain death, the number of harvested organs, the reasons why patient’s relatives refused to donate organs, and the number and specialty of the independent medical specialists who reviewed the case. Because of the observational and retrospective nature of the study, the University Research Deputy did not ask for the approval of the Ethics Committee.

During the last two years, we have established new Brain Death Identification Units (BDIU) in cities without previous background and trained their team of ICU nurses and coordinators. Therefore, our main activity in Tehran has moved to other OPUs. This made it possible to collect data from other centers too.

## Results

A total of 874 ICU admitted patients with severe brain injury (Glasgow coma score <7) in the hospital of origin had been introduced to our center and visited by the coordinator team from April 2007 to April 2012. Of these, 412 (47%) were excluded from the study for unsuitability for donation according to the medical protocols (n=205; 23.4%), not fulfilling the brain death criteria (n=66; 7.5%), they were found death before interviewing with their relatives (n=39; 4.4%), lack of facility management and logistics in the hospital of origin (n=12; 1.3%), lack of suitable recipients (n=7; 0.8%), absence of patients’ relatives (n=19; 2.2%), and for legal or suspicious issues regarding patient’s death.

**Figure 1:**
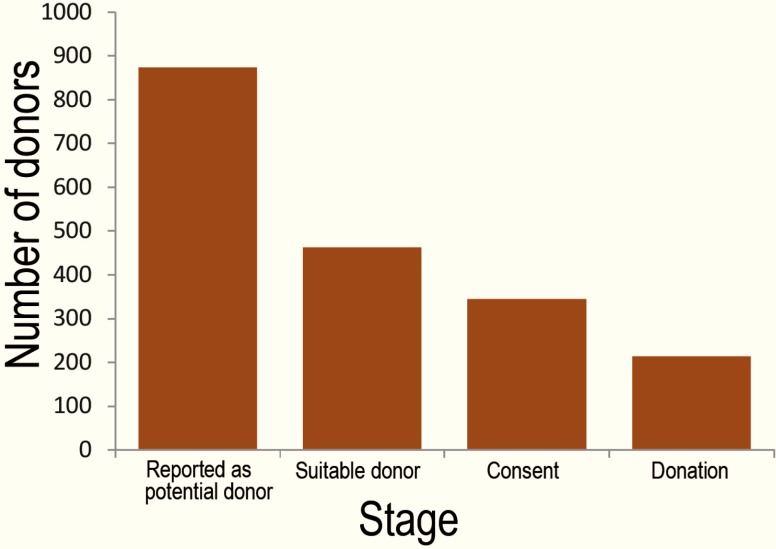
The number of donors at different stages from identification to donation

The families of the remaining cases (n=462) had been interviewed and 343 (74.2%) gave consent to donate their patients’ organs. During the process of supervision by our OPU, another 130 cases failed to donate (Fig 1) the main reasons of which were disagreement with the diagnosis of death and waiting for a miracle to happen.

**Figure 2:**
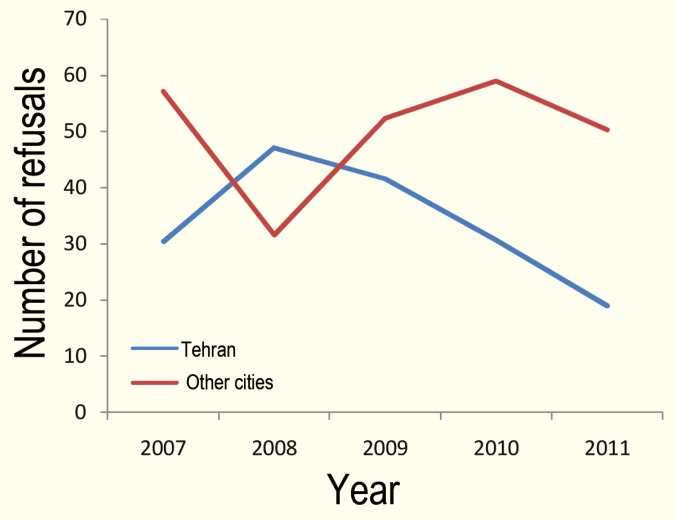
Number of refusals in Tehran compared to other cities without OPU, from 2007 to 2011

The demographic data of 213 remaining cases are summarized in Table 1. The mean±SD age of the patients was 29.8±13.2 years; the male/female ratio was almost 2 (141/72). The most common cause of brain death was head trauma due to traffic collisions (n=120; 56.3%) and cerebrovascular accidents (n=40; 18.8%). The refusal rate from 2007 to 2011 has decreased respectively, from 30.4% to 20% in Tehran, and from 57.1% to 51.6% in other cities (Fig 2). The overall refusal rate was 25.8%. The number of donors with organ retrieval increased from 20 in 2007 to 74 in 2011. The mean number of organs retrieved was 3.6.

**Table 1:** Demographics of dead-brain donors

		2007	2008	2009	2010	2011
Permission to donate % (absolute ratio)	Tehran	70 (32/46)	53 (37/70)	59 (38/65)	69 (68/98)	80 (30/37)
	Other cities	43 (3/7)	68 (13/19)	48 (10/21)	41 (16/39)	48.4 (79/159)
Cause of brain death						
Traffic collisions Brain hemorrhage Brain tumors Others		8 5 2 5	20 6 1 8	21 5 3	28 11 5 11	38 17 3 16
Mean±SD age (yrs)		33±13	28±13	28±12	31±12	30±15
Mean±SD time from patient admission to ICU to primary diagnosis of brain death (d)		4.1±6.2	2.32±2.3	3.1±2.5	3.09±3.6	2.88±3.2
Mean±SD time from the diagnosis of brain death to OPU		0.46±1.9	1.61±1.1	2.1±2	1.88±1.4	1.43±1.37
Mean±SD time from OPU admission to harvest		0.26±0.45	0.29±0.52	0.21±0.41	0.46±0.57	0.88±0.83
Mean number of harvested organs		3.8	3.6	3.6	3.7	4.0
Mean number of transplanted donors		3.7	3.4	3.6	3.6	3.6

## Discussion

One of the main concerns of all transplantation teams is understanding of relatives of a potential dead-brain donor of the nature of the brain death and the opportunity arisen to save other patients' lives by their appropriate actions and timely decision. Refusal to give consent for organ donation for this problem is still common in many countries including Iran [8-10]. In another OPU in Tehran, the refusal rate was 74% in 2009 [6]. What is important is the improvement in the rate of giving consent for solid organ donation. Saviozzi, *et al*, reported a reduction in refusal rate from 46.4% in 2001 to 19.5% in 2009 and concluded that the presence of experienced, committed health care personnel is mandatory to increase the available organ donor pool. Our study confirmed such a decrease and the role of experienced staff in OPU too [11]. On the other hand, general public education is necessary to persuade them to understand their responsibility for meeting the transplantation needs of the community, as it has already met for blood donation; also they should be informed of the meaning of brain death as clear as possible. Arjmand, *et al*, in their case-control study on 178 donor cardholders and the same number of a control participants showed that inadequate knowledge about donation and transplantation was the main reason for refusal to donate organ and tissue [12]. We need programs to sensitize the general population aiming at better acceptance of BDD process. This was confirmed by fluctuations in the refusal to consent rate in cities other than Tehran, despite the absolute rise in the number of donors (Fig 2); the nadir of graph was when some cities, without any background, were added to our OPU system. However, continuous support of an experienced team and the perseverance in repeated visits of all potential donors by the team members have increased the number of donation in spite of high refusal rates in cities other than Tehran. Another striking finding was the high percentage of donor pool from traffic collisions victims who had a mean age of 30 years. This was comparable to other studies from Iran and other developing countries; this emphasizes the necessity for a quick response of the team and widespread education [6, 7, 13]. Furthermore, the decrease in time between the patient admission to ICU and making the primary diagnosis of brain death by the first EEG taken by the coordinator team, is a promising finding which supports the positive impact of training and expertise plus quick response of the personnel for future planning.

Identification of potential donors by the ICU staff, which took place in this study, and reporting them to our organ procurement organization (OPU) resulted in taking consent from 53% of suitable donors. This rate was 80% in the study by Khoddami Vishteh, *et al* [6]. One of the main reasons for the difference observed between these two studies may be due to the centers recently joined to our center; the new centers did not have any background on the subject and if we could have had more training courses and case studies, the rate would be increased.

In conclusion, like other studies, we found that public belief has an important role in their refusal rate; presence of experienced coordinators would be very important in getting the consent from families; they can provide understandable answers to family members’ questions; their help is paramount even in areas without previous background in BDD.
